# Evaluation of the Diagnostic and Predictive Significance of Postoperative C-Reactive Protein to Transferrin or Albumin Ratio in Identifying Septic Events Following Major Abdominal Surgery

**DOI:** 10.3390/jcm14124341

**Published:** 2025-06-18

**Authors:** Taxiarchis Konstantinos Nikolouzakis, Athanasios Alegakis, Maria Niniraki, Marilena Kampa, Emmanouel Chrysos

**Affiliations:** 1 Department of General Surgery, University General Hospital of Heraklion, 71110 Heraklion, Greece; chrysose@uoc.gr; 2 Department of Forensic Sciences and Toxicology, Faculty of Medicine, Crete University, 71003 Heraklion, Greece; alegkaka@uoc.gr; 3 Laboratory of Experimental Endocrinology and Clinical Immunology, University Hospital of Heraklion, 71110 Heraklion, Greece; mniniraki@gmail.com; 4 Laboratory of Experimental Endocrinology, School of Medicine, University of Crete, 70013 Heraklion, Greece; kampam@uoc.gr

**Keywords:** C-reactive protein to albumin ratio, C-reactive protein to transferrin ratio, transferrin, C-reactive protein, surgical site infection, biomarker

## Abstract

**Background/Objectives:** Postoperative septic events represent a major paramevter of morbidity and mortality following major abdominal surgery. Early identification and prediction can have a major impact on clinical management, reduction of hospitalization costs, and restriction of irrational use of antibiotics. For this purpose, two novel biomarkers (C-reactive protein to albumin or transferrin ratios, CAR and CTR, respectively) were evaluated. **Methods**: A combined retrospective and prospective study of 200 patients who underwent elective or emergency open abdominal surgery was performed. Patient demographics, emergency status, type of operation, and white blood cell (WBC) count, serum albumin (ALB), serum transferrin (TRF), and CAR-CTR were evaluated. Multiple-way ANOVA was utilized. Multiparametric and logistic regression analyses were performed for each confounder. Receiver operating characteristic (ROC) curve analysis and corresponding diagrams of sensitivity vs 1-specificity were applied for CAR and CTR in postoperative days 2 and 3. **Results**: WBC number had no predictive significance in septic event identification (*p* = 0.461), while postoperative CAR, CTR, ALB, TRF, BMI, and emergency status were significantly correlated (*p* < 0.001). At postoperative day 2, a CTR of 9.48 and a CAR of 4.14 have 75.9% and 70.4% specificity and 86% and 87.7% sensitivity, respectively. At postoperative day 3, a CTR of 8.89 and a CAR of 4.25 have 74.1% and 79.6% specificity and 87.7% and 86% sensitivity, respectively. **Conclusions**: Early identification of postoperative septic events may significantly facilitate decreasing postoperative morbidity and mortality. Both CAR and CTR displayed significant predictive ability in identifying patients prone to developing postoperative septic events, highlighting their significance in everyday clinical practice.

## 1. Introduction

Postoperative septic events, such as surgical site infection (SSI) and anastomotic leak (AL), are major determinants of morbidity and mortality following abdominal surgery regardless of whether an open or laparoscopic approach has been followed [1,2], even though open operations are more prone to develop SSI [1]. Moreover, emergency status [3], socioeconomic status [4], and the underlying risk factors of each patient (such as obesity, hyperglycemia, and smoking) [5,6] seem to also affect SSI development. Apart from the clinical consequences (morbidity, prolonged length of hospital of stay, and development of resistant microbial strains), SSIs result in a significant economic burden for the healthcare systems and patients [7]. It is estimated that patients with a SSI may have higher hospitalization costs than those without it (EUR 16.685 or USD 19.703 and EUR 11.235 or USD 13.267, respectively) [8]. The same observations apply for ALs. However, their prevalence and consequences seem to vary according to the site of the failed anastomosis [9,10]. AL refers to a leakage at a surgical join in the bowel whose clinical significance may range from mild symptoms such as fever, abdominal discomfort, with mild leukocytosis, and increase in inflammatory biomarkers to peritonitis, depending on the site of anastomosis (intraperitoneal leak tends to have more clinically pronounced symptoms as opposed to retroperitoneal), to the presence of enteric content inside the lumen (for example, in cases of very low coloanal anastomoses, the current trend is to create a diverting ileostomy in order to prevent the development of clinically significant leaking in case of an anastomotic failure). For this reason, several prognostic and predictive tools that utilize pre and postoperative clinical data and health risk factors have been proposed [11,12,13,14,15]. Despite their overall good performance though, they seem to have poor diagnostic performance. For this reason, a number of studies tried to validate the diagnostic and/or predictive significance of biomarkers in order to early identify postoperative septic events. For instance, high levels of circulating procalcitonin (PCT) and C-reactive protein to albumin ratio (CAR) have been proposed as indicators of SSI and AL in colorectal surgery [16,17,18,19,20], while increased levels of peritoneal cytokines tumor necrosis factor (TNF) and interleukin 6 (IL-6) have also been proven to correlate with AL [21] and SSI [22], even though CAR, PCT, TNF, and IL-6 seem to outperform C-reactive proteins (CRPs) for these purposes [16,19,23]. The reason for choosing these biomarkers (or their combination) lies in the fact that surgical operations (especially open ones) are significant drivers of systematic stress responses affecting the metabolism as well as the neuroendocrine and immune systems [24]. If surgical stress is prolonged, its effects may propagate leading the organism to enter hypercatabolism (with substantial decrease in circulating albumin-ALB), impairment of wound healing mechanisms, increase in acute-phase proteins (such as PCT and CRP), and ultimately systematic inflammatory response syndrome (SIRS), in which unregulated production of pro-inflammatory cytokines (TNF/IL-6) and deregulated immune responses are observed [25,26]. However, routine assessment of PCT, TNF, and IL-6 may be impeded by the substantial costs involved and the scarcity of clinical laboratories equipped to perform these tests. This is especially relevant for secondary care hospitals and mid- to low-income territories where complex or expensive tests cannot be implemented in everyday clinical practice. On the contrary, there is evidence that other more cost-effective acute-phase proteins, such as transferrin (TRF), may have a strong correlation with microbial infection, inflammation, and nutritional status [27,28,29,30]. TRF is a key glycoprotein in iron metabolism, being crucial for iron transport and homeostasis [31]. It is a monomeric glycoprotein with a molecular weight of approximately 80 kDa, consisting of two homologous lobes, the N-lobe and the C-lobe, each capable of binding one ferric ion (Fe^3+^), allowing TRF to transport iron ions in the bloodstream [32]. It is well identified that TRF plays a crucial role in maintaining iron homeostasis by binding free iron in the blood, thus reducing the availability of free iron for catalyzing harmful free radical formation while also affecting innate immune response. In states of infection and inflammation, TRF levels can decrease, limiting iron availability to pathogens and thereby inhibiting their growth [33]. However, TRF levels may change in the presence of chronic disease anemia or iron deficiency anemia (low vs. elevated levels, respectively). Interestingly, it has been shown that in states of inflammation and bacterial infection TRF may exhibit earlier changes and higher sensitivity than ALB [34,35]. For this reason, we aimed to evaluate the diagnostic and predictive efficacy of the CRP to TRF ratio (CTR) against CRP and CAR for SSIs and ALs. To do so, a combined retrospective and prospective study was performed. In order to test the initial assumption that patients with a SSI had different CAR than those without, a retrospective group of patients was formed using predetermined inclusion and exclusion criteria. After showing that a difference in CAR values between the two groups was present, we formed a prospectively studied group using the same inclusion and exclusion criteria in order to evaluate and set the best cut-off values of the predictive significance of CAR while also testing the same parameters for CTR.

## 2. Materials and Methods

### 2.1. Patients

A combined retrospective and prospective study was conducted between 2020 and 2024, which included 200 patients (114 males and 86 females). The retrospective arm involved 89 randomly chosen patients, using hospital records, who underwent emergency or elective major open abdominal surgery at the Department of General Surgery of the University Hospital of Heraklion between 1 January 2020 and 1 December 2023. The same inclusion and exclusion criteria were used for the prospective group. Patients with SSIs were identified using a prospectively maintained database of positive wound cultures and were further cross-referenced with hospital records. For the prospective arm of the study, all patients who were submitted to an emergency or elective major open abdominal operation between 1 December 2023 and 1 July 2024 were evaluated for their potential to be included. In total, 128 were initially evaluated, however, only 111 were included, since four patients refused to participate, five patients died before completing the study, three were re-operated on at 48 h, and another five had incomplete data.

### 2.2. Establishment of Postoperative Septic Event Diagnosis

For the establishment of a postoperative septic event diagnosis (including SSI), guidelines from the National Healthcare Safety Network, which is part of the Centers for Disease Control and Prevention, were implemented [36]. Moreover, the infection was confirmed with microbiological evidence of positive wound cultures. Cultures isolating *Staphylicoccus epidermidis* as the only pathogen were not interpreted, except if Methicillin-resistant *Staphylococcus epidermidis* (MRSE) was identified. Our department does not regularly screen patients for *Methicillin-resistant Staphylococcus aureus*. Therefore, any isolation of this pathogen is discussed separately with the infections control committee in order to plan a surveillance protocol. Based on this, SSI monitoring was based upon active, patient-based, and prospective surveillance. For the diagnosis of an intra-abdominal abscess, a computed tomography was used. Concurrent and post-discharge surveillance methods were used to detect postoperative septic events following inpatient and outpatient operative procedures for a 30-day period after the operation for all procedure types. For the outpatient surveillance, a revised Macefield’s questionnaire for SSIs was translated in Greek and used [37], along with a predetermined clinical examination by a surgeon working for the Department of General Surgery at postoperative day 15.

### 2.3. Study Protocol

After an initial identification of the predictive significance of CAR in the retrospective group, cut-off points were established and used as references for the prospective group in order to estimate the diagnostic potential of both CAR and CTR. In a final step, in order to evaluate the diagnostic performance of both CAR and CTR in the early detection of wound infection as opposed to clinical evaluation, 40 patients were randomly chosen and evaluated by senior surgeons for signs and symptoms of wound infection at postoperative day 2 and 3. In the case that a patient’s CAR and/or CTR values were at or above the cut-off points or if the surgeon suspected a wound infection, wound cultures were retrieved. Their estimation of the presence of wound infection was recorded and compared against the diagnostic accuracy of CAR/CTR. The protocol for this study has been approved by the Ethics Committee for Patients and Biological Material of the University Hospital of Heraklion, Greece (registration number 18/07-12-2023; date: 7 December 2023). All participants signed an informed consent agreement. All samples generated by this study were anonymized, and personal data were managed according to the EU General Data Protection Regulation (GDPR).

### 2.4. Inclusion Criteria

All patients who were submitted to a major open abdominal operation as defined by Courtney et al. [38,39] were considered as potential candidates. In short, a major abdominal operation should be defined as any intra-peritoneal operation with no primary involvement of the thorax that implements either luminal resection and/or resection of a solid organ associated with the gastrointestinal tract. Consecutive evaluation of CRP and ALB for the first three postoperative days along with a positive wound culture had to be present for a patient to be included in the retrospective arm.

### 2.5. Exclusion Criteria

Exclusion criteria for both arms were as follows:

(I) Lack of microbiological evidence of SSI,
(II) Refusal of the patient to attend the study,
(III) Reoperation prior to postoperative day 3,
(IV) ALB infusion either preoperatively or within 2 postoperative days,
(V) Known autoimmune disease under treatment or not,
(VI) Incomplete laboratory data,
(VII) Anemia of chronic disease.

### 2.6. Blood Sampling

Peripheral blood samples were collected at predetermined time points preoperatively, at 24, 48, and 72 h postoperatively. All blood samples were stored in 5 °C until processing within 48 h from sampling.

### 2.7. Data Collection

The data of interest included demographic characteristics (age, sex, and body mass index—BMI), operative data (emergency or elective operation, type of operation), laboratory data (postoperative white blood cells—WBCs, CRP, ALB, and TRF in 24/48/72 h), and comorbidities (namely anemia of chronic disease, diabetes mellitus type 1 or 2, chronic liver disease, and chronic renal disease).

### 2.8. Control Group

The control group was constituted by 81 individuals with no clinical or microbial indication of SSI who were submitted to major abdominal operation.

### 2.9. Statistical Analysis

All statistical analyses were conducted using IBM SPSS Statistics 14. Descriptive statistics were used to summarize demographic and clinical characteristics, with continuous variables expressed as means ± standard deviations (SD) or medians with interquartile ranges (IQR) depending on their distribution, and categorical variables presented as frequencies and percentages. The normality of continuous data was assessed using the Shapiro–Wilk test. For comparative analyses, preoperative albumin levels were categorized into five clinically relevant groups (<2.5, 2.5–3, 3–3.5, 3.5–4, and >4 g/dL) and their association with surgical site infection was evaluated using Pearson’s chi-square test, supplemented by Fisher’s exact test when expected cell frequencies were below five. The strength of these associations was quantified using Cramér’s V, and a post-hoc power analysis was performed in G*Power 3.1 using the observed effect size (V = 0.20), an alpha of 0.05, and 4 degrees of freedom to determine the study’s statistical power. Additional between-group comparisons employed Mann–Whitney U tests for non-normally distributed continuous variables and chi-square or Fisher’s exact tests for categorical variables as appropriate. Multiple logistic regression analysis was conducted to identify independent predictors of postoperative septic events, including the categorized albumin levels as potential predictors. Due to computational constraints in the logistic regression model, the CTR variable was scaled by multiplying by 100 to ensure proper model functioning. All regression results are reported as odds ratios (OR) with 95% confidence intervals (CI). The diagnostic performance of the CAR and adjusted CTR for predicting postoperative septic events was evaluated using receiver operating characteristic (ROC) curve analysis, with the area under the curve (AUC) calculated to assess accuracy and optimal cutoff values determined to maximize both sensitivity and specificity. For all analyses, a two-tailed *p*-value < 0.05 was considered statistically significant.

### 2.10. Theory/Calculation

The predictive power of CTR and CAR hinges on their reflection of two interrelated pathophysiological processes: systemic inflammation and metabolic stress. While CRP is a well-established acute-phase reactant synthesized in response to IL-6 and TNF-α, its ratio to ALB or transferrin amplifies its diagnostic utility by integrating nutritional and iron-homeostasis derangements. Transferrin, a negative acute-phase protein, declines during inflammation due to cytokine-mediated hepatic suppression, while its role in iron sequestration limits microbial growth. This dynamic renders CTR that is particularly sensitive to early infection, as transferrin’s shorter half-life (~8 days) compared to ALB (~21 days) may capture inflammatory shifts more rapidly. Theoretically, CTR’s diagnostic advantage over CAR arises in contexts of hypoalbuminemia (e.g., malnutrition, exogenous ALB administration), where ALB’s fluctuations may obscure the true inflammatory burden.

Calculationally, the ratios were derived as follows:

$$\mathrm{CAR}=\frac{CRP\left(\frac{mg}{dL}\right)}{Albumin\left(\frac{g}{dL}\right)}\;\mathrm{and}\;CTR=\frac{CRP(\frac{mg}{dL})}{Transferrin(\frac{mg}{dL})}$$

To address computational constraints in logistic regression, CTR was scaled by 100 (CTR × 100), preserving its predictive validity while ensuring model stability. ROC curve analysis optimized cutoff values by maximizing Youden’s index (sensitivity + specificity – 1), with AUC comparisons confirming CTR’s non-inferiority to CAR. This approach bridges theoretical pathophysiology (acute-phase responses) with clinical applicability, offering a quantifiable metric for early septic risk stratification.

## 3. Results

### 3.1. Patient Characteristics

In total, 200 patients were included in the study, 89 in the retrospective arm and 111 in the prospective, 86 of whom were females and 114 were males. For the retrospective group, the mean age was 63.9 years, while for the prospective group it was 66.6. Overall, 102 patients were submitted to an elective operation (51%), while the mean length of hospital stay was 11.1 days. Demographic characteristics are presented in Table 1. The type and number of operations for each group are presented in Supplementary Table S1. Chronic diseases that may contribute to SSIs along with cancer rates for each group are presented in Supplementary Table S2. It is shown that apart from hypertension, the rest of the chronic diseases and cancer rates exhibit no statistically significant difference among the two groups. The incidence of postoperative septic complications is presented in Table 1. In detail, it is evidenced that 48.6% and 69.7% of the prospective and retrospective groups accordingly had such a complication. The most common complication was wound infection (59.5%), followed by wound dehiscence (18%). Moreover, the majority of patients suffered from one septic complication (30.6% and 35.5%, respectively). A detailed description of the incidence for each septic event per type of operation is given in Supplementary Table S3.

### 3.2. Establishment of Postoperative Septic Event Diagnosis

Out of the 111 patients that were included in the prospective arm, 57 developed a postoperative septic event (51.4%). In detail, 57 developed wound infection (51.4%), 19 developed wound dehiscence (17.1%), 12 developed an intrabdominal abscess (10.8%), and 9 developed an anastomotic/stump or suture line leak (14.1%). Regarding wound cultures, *Staphylicoccus epidermidis* was isolated from five patients, and these cultures were not considered as positive (no MRSE was isolated), *Escherichia coli* was isolated in 41 specimens (71.9%), Enterococcus spp. in 33 (57.9%), *Streptococcus* spp. in 28 (49.1%), and *Candida* spp./*Klebsiella* spp. in 19.3% and 24.6%, respectively. Out of the 57 patients, 49 were diagnosed while hospitalized and 8 as outpatients. For these eight patients, the revised Macefield’s questionnaire had at least two positive answers for the signs and symptoms that are indicative of a wound infection and seven had at least two positive answers for wound care interventions.

### 3.3. Evaluation of the Correlation Between Patient Characteristics, Laboratory Tests, and Postoperative Septic Events

- Laboratory tests

As presented in Table 2, the comparison of WBCs, ALB, and TRF values between patients with or without postoperative septic complications did not reach statistical significance. On the contrary, CRP, CAR, and CTR measured at postoperative day 1, 2, and 3 proved to have a statistically significant difference (*p* < 0.001) between patients with or without postoperative septic complications and as in a total group of measurements.

- Correlation of preoperative ALB levels with SSI incidence

Correlation of preoperative ALB levels of the prospectively observed patients with SSI incidence (ALB was not always measured in the preoperative setting among patients of the retrospective group) revealed a trend toward statistical significance in the association between preoperative albumin levels and surgical site infection (χ^2^(4) = 4.27, *p* = 0.054). The highest incidence of surgical site infection was observed in the group with albumin levels below 2.5 g/dL, with progressively lower rates seen in higher albumin categories. The effect size for this association was small to moderate (Cramér’s V = 0.20). Post-hoc power analysis indicated the study had only 35% power to detect the observed effect size, suggesting a substantial risk of type II error in these findings (Supplementary Table S4).

- Multiparametric analysis

Following a multi-parametric analysis for binary and continues variables, it was shown that male sex, the prospective nature of the study, emergency operations, BMI, CRP at postoperative day 2 (Post Op CRP DAY2), ALB at postoperative day 2 (Post Op ALB DAY2), CAR at postoperative day 2 (CAR DAY 2), CTR at postoperative day 2 (CTR DAY 2), and CTR at postoperative day 2 multiplied by 100 (CTR DAY2 × 100) were strongly correlated with the event of postoperative septic complications reaching statistical significance (Table 3).

- Multiple logistic regression analysis

Incidences of postoperative septic complications after abdominal surgery were tested for predictive explanatory factors using multiple logistic regression analysis for binary and continuous variables. It was shown that female sex and high postoperative ALB values at D2 had a strong significance in protecting against postoperative septic complications, while postoperative CTR × 100 and CAR at postoperative days 2 and 3 had a strong negative impact upon septic postoperative complications (Table 4).

### 3.4. Predictive Value of CRP, CAR, and CTR for Postoperative Septic Complications

**Figure 1:**
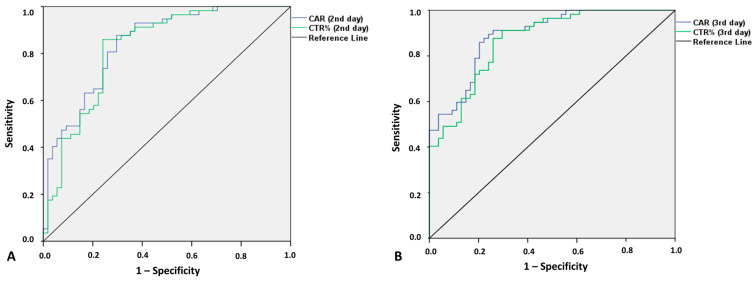
ROC curve analysis for CAR and CTR at the second and third postoperative day. Chart (**A**) represents ROC curves for CAR and CTR at the second postoperative day. Chart (**B**) represents ROC curves for CAR and CTR at the third postoperative day. (C reactive protein to albumin ration CAR, C reactive protein to transferrin ratio CTR, Receiver of characteristics ROC).

In order to evaluate the predictive value of CRP, CAR, and CTR for the identification of postoperative septic complications, a ROC curve analysis was performed and the best sets of sensitivity/specificity were identified. CRP as a standalone index showed an AUC of 0.722 (95%CI 0.65–0.79) at day 1, 0.822 (95%CI: 0.76–0.88) at day 2, and 0.839 (95%CI: 0.78–0.89) at day 3. As shown in Figure 1, CTR DAY2 × 100 had an AUC of 0.825 (95% CI 0.745–0.904) while CAR DAY 2 had an AUC of 0.848 (95% CI 0.794–0.902). CTR DAY3 × 100 had an AUC of 0.868 (95% CI 0.803–0.932), while CAR DAY 2 had an AUC of 0.890 (95% CI 0.832–0.948). Given that both CAR and CTR outperformed CRP after ROC analysis for both day 2 and day 3, further analysis of the cut-off points was made only for CAR and CTR. For CAR at postoperative days 2 and 3, the best set of sensitivity and specificity was found at 4.14 (sensitivity 0.877, specificity 0.704) and 4.26 (sensitivity 0.86, specificity 0.796), respectively, while for CTR (multiplied by 100) at postoperative days 2 and 3, they were found at 9.48 (sensitivity 0.860, specificity 0.759) and 8.89 (sensitivity 0.877, specificity 0.741), respectively.

### 3.5. Correlation Between Examiner’s and CAR/CTR Diagnostic/Predictive Performance

**Figure 2:**
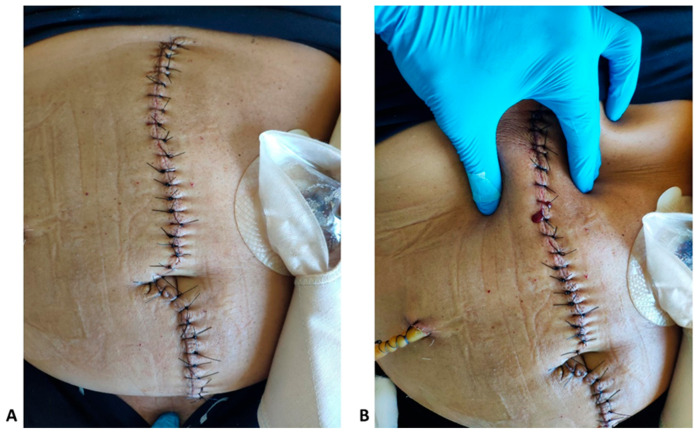
(**A**) Photographic documentation of a clinically considered clean wound at postoperative day 3 (Hartmann’s sigmoidectomy). (**B**) Photographic documentation of the same wound. Due to the positive CAR/CTR, thorough clinical evaluation of the wound revealed a haemoserous exudate from which the wound culture isolated *E. coli, Enterococcus* spp. and *Candida* spp.

Out of the 40 patients that were included, 26 (65%) were found to have a wound infection proven by wound culture. Regarding CAR/CTR performance, both ratios were able to correctly identify 25 out of 26 wound infections (96.2%) starting at postoperative day 2, while CAR was falsely positive two times, resulting in *Staphylicoccus epidermidis* isolation. On the contrary, clinical examination resulted in three true positive identifications of wound infection (11.5%). However, these three cases that were correctly identified by clinical examination were also identified by CAR or CTR. An example of a falsely negative wound infection is depicted in Figure 2.

## 4. Discussion

Postoperative septic events such as SSI and AL are two major drivers of morbidity and mortality following major abdominal operations leading to prolonged hospital stay and a net increase in hospitalization cost [40,41]. It is estimated that 0.5–11% of all surgical patients will develop an SSI [40] and 3–19% of the patients with an anastomosis will develop a clinically relevant AL [42]. In order to address this problem, several organizations proposed a number of bundles of pre and intraoperative measures [43]. According to our data, 51.4% of the prospective and 30.3% of the retrospective arms developed a postoperative septic event. No significant difference between the AL and intrabdominal abscess formation rate was detected. The higher percentage of wound infections than that described in the literature may be partially explained by the fact that, to a certain extent, daily wound handling was not performed using strict sterile conditions (such as sterile gloves, sterile drapes, and two examiners per patient). Ιn coordination with the infection control committee of our hospital, our department adopted certain perioperative measures in order to address the high incidence of wound infections. However, improvements in outpatient care over the past five years facilitated earlier hospital discharge, even in cases of moderate wound infections. Interestingly, as indicated by the multi-parametric analysis, none of well-known risk factors for SSI/AL were found to be statistically important. The only parameters that were found to be strongly related with SSI/AL were male sex, emergency operations, BMI, CRP, ALB, CAR, and CTR. Male sex is controversially discussed as a risk factor for SSI after abdominal surgery, with ample evidence supporting both standpoints [44,45,46]. Emergency status of an operation is a well-accepted risk factor for postoperative SSI/AL [2], for which numerous perioperative protective measures have been proposed, such as intraoperative wound protection, wound irrigation with saline, anti-bacterial solution, or a diluted povidone-iodine solution, perioperative normothermia, euglycemia, prophylactic negative pressure wound therapy, and others [47,48,49,50]. Two more well-identified risk factors are obesity (in our case expressed as high BMI) and malnutrition (in our case expressed as low albumin levels) [51,52], for which there is evidence that both share the same mechanisms to a certain extent (dysregulated immune function, impaired tissue oxygenation and regeneration, altered microbial environment, and prolonged inflammatory state). In detail, it is proved that in obesity, chronic low-grade inflammation is present since adipose tissue produces pro-inflammatory cytokines (e.g., TNF-α, IL-6), chemokines (monocyte chemoattractant protein (MCP1/CCL2) [53], and pro-inflammatory fatty acids [54], leading to systemic inflammation. This chronic inflammation drives alterations in leukocyte number and phenotype, thereby expanding the inflammatory environment within adipose tissue beds. It has been recently described that intestinal B cell homeostasis and regulation and function are significantly impaired due to obesity, resulting in a net reduction in immunoglobulin (Ig) A + B cells and IgA producing plasma cells in mesenteric lymph nodes, and secretory IgA antibody concentrations in colon in DIO mice [55]. In addition, due to chronic systemic inflammation, insulin signaling pathways are impaired via mechanisms such as the serine phosphorylation of the insulin receptor and insulin receptor substrate-1 through the activation of IkB kinase, c-Jun N-terminal kinase and protein kinase C [56]. This results in macrophage dysfunction, while hyperglycemia, due to insulin resistance, can further impair normal function of neutrophils and T-cells [57]. On the other hand, malnourished patients have protein–energy malnutrition, which reduces the production of immunoglobulins, cytokines, and complement proteins. In addition, deficiencies in vitamins (e.g., A, C, and D) and minerals (e.g., zinc, selenium) further weaken immune response and antioxidant capacity [58]. The current study identified a clinically relevant trend toward higher surgical site infection rates among patients with lower preoperative albumin levels, although this association did not reach conventional statistical significance. This finding aligns with established literature, demonstrating the relationship between hypoalbuminemia and postoperative complications. However, the low statistical power of 35% significantly limits confidence in these results, as the study had a 65% probability of failing to detect a true effect of this magnitude. The small to moderate effect size further suggests that while albumin may serve as a potential marker for infection risk, its predictive value might be limited in isolation. Regarding the common impairment of tissue oxygenation and regeneration, excess adipose tissue has poor vascularization, leading to hypoxia in wound areas, which impairs fibroblast activity, collagen deposition, and angiogenesis, which are critical for wound healing. Malnutrition, though, exhibits delayed wound healing through the lack of protein and essential nutrients that impairs fibroblast proliferation, collagen synthesis, and angiogenesis, while anemia leading to impaired oxygen delivery is also observed. Energy deficits further reduce the availability of ATP needed for cellular repair and regeneration. In our series of patients, the preoperative nutritional status was evaluated only in the prospective arm, given that this was not included in the inclusion/exclusion criteria. However, microbiome alterations are also a common ground in obesity and malnutrition since obesity can alter skin and gut microbiomes, increasing colonization with pathogenic bacteria, while malnutrition can lead to gut barrier dysfunction, leading to bacterial translocation and systemic infections [59]. Regarding CRP, CAR, and CTR, all three components (CRP, ALB and TRF) are identified as acute-phase proteins [60,61]. CRP is a highly conserved acute-phase protein primarily produced by hepatocytes in response to interleukin-6 (IL-6), interleukin-1β (IL-1β), and tumor necrosis factor-α (TNF-α) stimulation during inflammation. IL-6 binds to its receptor (IL-6R), triggering activation of the JAK/STAT pathway in hepatocytes. Phosphorylated STAT3 translocates to the nucleus and binds to specific enhancer regions in the CRP gene promoter, initiating transcription [62]. While hepatocytes are the main source, extrahepatic production has been reported in small amounts in tissues such as adipose tissue, lungs, renal epithelial cells, and vascular smooth muscle cells [63,64]. As a critical component of the innate immune system, CRP serves as both a biomarker and a modulator of inflammatory processes. It has been proven that CRP levels begin to rise 6–8 h after the onset of an inflammatory stimulus, reaching a peak at 48 h. Once the inflammatory stimulus resolves, its levels decline rapidly given the relative short half-life (approximately 19 h) [65]. TRF is a glycoprotein predominantly synthesized by hepatocytes in the liver that plays an important role in iron metabolism since it is crucial for iron transport and homeostasis [31]. The expression of the TRF gene is regulated by transcription factors such as hepatocyte nuclear factor-4α (HNF-4α), which plays a key role in maintaining baseline transferrin expression in hepatocytes and the hypoxia-inducible factor-1α (HIF-1α), which under hypoxic conditions, upregulates TRF synthesis in order to facilitate iron transport for erythropoiesis [66]. Owing to its role in maintaining iron homeostasis, TRF regulates the availability of free iron, and therefore free radical formation, while also affecting innate immune response. In states of infection and inflammation, TRF levels decrease, limiting iron availability to pathogens and thereby inhibiting their growth [33]. Pro-inflammatory cytokines (IL-6 and TNF-α) are known downregulators of TRF production [67]. In a normal state, its plasma half-life is approximately 8 days. However, TRF levels may change in the presence of chronic disease anemia or iron deficiency anemia (low vs elevated levels respectively). ALB is the most abundant protein in human plasma, constituting approximately 50–60% of the total plasma protein content. It is synthesized exclusively by hepatocytes, and its production is tightly regulated by various factors, including nutritional status, hormonal signals, and inflammatory responses [68]. Infection triggers a complex immune response that can significantly affect ALB synthesis and function. During an acute-phase response acute-phase proteins such as CRP, fibrinogen, and serum amyloid A (SAA) are produced, while ALB synthesis is suppressed [69]. This downregulation serves as a protective mechanism aiming at prioritizing the production of proteins that are essential for immune defense and tissue repair. This occurs via the activation of signaling pathways such as the JAK/STAT pathway, which leads to changes in gene expression that favor the synthesis of acute-phase proteins while inhibiting ALB gene expression. TNF-α and IL-1 contribute to the suppression of ALB production by direct action on hepatocytes. TNF-α, in particular, can activate nuclear factor kappa B (NF-κB) signaling, which is involved in the inflammatory response and can directly suppress ALB synthesis by interfering with the transcriptional machinery in hepatocytes [70]. What is more, pro-inflammatory cytokines seem to be able to stimulate ALB breakdown by the liver and other tissues, further reducing circulating ALB levels [69]. Interestingly, it has been shown that in states of inflammation and bacterial infection, TRF may exhibit earlier changes and higher sensitivity than ALB [34,35]. For this reason and in conjunction with the nutritional status of a patient, our study showcased their significant role in predicting SSIs and ALs. Interestingly, it has been shown that plasma ALB and TRF, as opposed to plasma CRP [71], demonstrate clinically significant fluctuations, with their nadir presenting during the night [72,73,74]. For this reason, our samplings were always performed in the morning (except from the preoperative samplings that did not have a time sampling consistency due to the emergency cases). Although CRP is a well-established and sensitive acute-phase protein, our findings demonstrate that combining CRP with ALB or TRF improves its predictive performance for postoperative septic complications. While CRP alone yielded respectable accuracy—particularly on postoperative day 2 (AUC: 0.822, 95% CI: 0.76–0.88) and day 3 (AUC: 0.839, 95% CI: 0.78–0.89)—both CAR and CTR achieved higher AUCs during the same time points. Specifically, CAR reached an AUC of 0.848 and 0.890 on days 2 and 3, respectively, while CTR showed AUCs of 0.825 and 0.868. These improvements, although modest, suggest a meaningful prognostic value. This likely reflects their role as negative acute-phase proteins and indicators of nutritional and metabolic reserve, which as already mentioned, are known to modulate the systemic response to surgical stress and infection. Thus, it is made clear that clinicians should not only take into account CRP, but rather CAR and/or CTR in order to extract safer conclusions regarding the probability of developing an SSI after a major abdominal operation. It is noteworthy that even though the predictive significance of CAR in certain abdominal operations has been exhibited, our study was the first to our knowledge to have used and proven the clinical significance of CTR in SSIs/Als after major abdominal surgery [75,76]. The addition of CTR in the clinical armamentarium is critically important because as our data indicate, a CTR value of 9.48 measured at postoperative day 2 achieved greater specificity (75.9%) than a 4.14 value of CAR (70.4%), while having almost the same sensitivity 86% and 87.7%, respectively. On the other hand, a CTR value of 8.89 measured at postoperative day 3 has 74.1% specificity and 87.7% sensitivity as opposed to a CAR value of 4.25 that demonstrates a 79.6% specificity and 86% sensitivity. Moreover, in critically ill and malnourished patients, clinicians may choose to administer human ALB solutions as intravascular volume expanders, especially in sepsis, where fluid resuscitation with crystalloids alone may be insufficient or even harmful (for example, in case of heart failure), or in combination with diuretics in order to address fluid overload and improve oxygenation by reducing pulmonary edema. In such cases, serum ALB will be falsely increased, leading to an inaccurate low CAR and therefore prohibiting its predictive capacity. However, serum TRF will not be affected by the exogenous administration of human ALB, allowing its use for predicting major septic events. Interestingly, both biomarkers significantly outperformed clinical evaluation in detecting septic events, even by senior surgeons. Their routine use could enhance early detection, enabling timely interventions, such as thorough wound care, early wound cultures, proper upscaling of antibiotics (in accordance to the local infection control committee guidance), early imaging (ultrasonography or computed tomography), or even early use of negative pressure wound therapy. This approach would reduce complications, improve rational use of antibiotics, prevent the emergence of resistant microbial strains, and lower healthcare costs. However, our study faces certain restrictions to drawing safe conclusions. To begin with, our sample size is relatively small. Therefore, even though we were able to discriminate between different septic complications (wound infections, SSIs, and AL), the relative numbers of each complication were small, and for this reason, no further analysis regarding possible predictive cut-off values was made for each complication. Moreover, the clinical significance was not always the same; a mild wound infection could be treated in the outpatient department, as opposed to severe infections, which require prolonged hospitalization. Next, even though our department does not have a special surgical focus that would restrict the range of operations, being a single center trial, we do not cover the full range of general surgery (for example advanced hepatobiliary, pancreatic, or esophageal surgery). In addition, the majority of the operations involved the alimentary tract (70.5%), and for this reason, no safe conclusions can be drawn regarding any possible predisposition of a certain type of operation to develop SSIs over another. Moreover, while preoperative ALB levels exhibited a clinically relevant trend of positive correlation with SSI incidence among patients with lower preoperative albumin levels, our group of patients was too relatively small to achieve significant power. Although this finding aligns with established literature demonstrating the relationship between preoperative hypoalbuminemia and postoperative complications [77,78,79], future studies with larger groups of patients are needed in order to achieve significant results. Another limiting factor is the lack of long-term surveillance given that our protocol followed up with our patients for 30 days and the absence of interventional measures for those patients who had high CAR and or CTR in order to evaluate if these biomarkers have a clinically significant impact on the clinical course. Finally, we did not estimate the intraoperative blood loss in order to include it in multiparametric analysis. However, it has to be noted that based on current evidence from various types of operations (general surgery [80], thoracic surgery [81], and gynecology [82]), excessive blood loss can be linked to postoperative SSIs. For these reasons, a larger study with a wider range of emergency and elective operations with longer follow up and more detailed perioperative data is needed.

## 5. Conclusions

Postoperative septic events remain a significant cause of morbidity and prolonged hospitalization following major abdominal surgeries. This study demonstrated that both CAR and the novel CTR are reliable and accessible biomarkers for early identification of such complications. CTR, in particular, showed predictive capabilities comparable to CAR, and in certain contexts—such as hypoalbuminemia or exogenous albumin administration—may offer superior clinical utility. These findings underscore the importance of incorporating cost-effective, routinely available biomarkers into postoperative surveillance protocols, especially in resource-constrained settings. Furthermore, both CAR and CTR significantly outperformed clinical evaluation alone, suggesting that their integration into daily surgical practice could facilitate earlier diagnosis, optimize antimicrobial stewardship, reduce healthcare costs, and ultimately improve patient outcomes. Future multicenter studies with larger and more diverse populations are warranted to validate these findings and explore stratified risk models based on individual complication types.

## Figures and Tables

**Table 1:** Demographic characteristics and mean length of hospital stay (in days) and presence of postoperative complications and the incidence of each complication. *p* values below 0.05 are highlighted in bold format (SD standard deviation, n number, R rate).

Type of Study								
		Prospective		Retrospective		Total		*p*
		N (%)		N (%)		N (%)		
Sex	Female	48 (55.8%)		38 (44.2%)		86 (43%)		0.94
	Male	63 (55.3%)		51 (44.7%)		114 (57%)		
Age groups	≤40	8 (66.7%)		4 (33.3%)		12 (6%)		0.68
	41–50	8 (53.3%)		7 (46.7%)		15 (7.5%)		
	51–60	17 (58.6%)		12 (41.4%)		29 (14.5%)		
	61–70	38 (61.3%)		24 (38.7%)		62 (31%)		
	71–80	27 (49.1%)		28 (50.9%)		55 (27.5%)		
	81+	13 (48.1%)		14 (51.9%)		27 (13.5%)		
Elective operation	Yes	62 (60%)		40 (39.2%)		102 (51%)		0.12
	No	49 (40%)		49 (60.8%)		98 (49%)		
Postoperative complications	No	54 (48.6%)		27 (30.3%)		81 (40.5%)		**0.002**
	Yes	57 (51.4%)		62 (69.7%)		119 (59.5%)		
	1	34 (30.6%)		37 (41.6%)		71 (35.5%)		
	2	10 (9%)		21 (23.6%)		31 (15.5%)		
	3	9 (8.1%)		4 (4.5%)		13 (6.5%)		
	4	4 (3.6%)		0 (0%)		4 (2%)		
Wound infection	No	54 (48.6%)		27 (30.3%)		81 (40.5%)		**0.009**
	Yes	57 (51.4%)		62 (69.7%)		119 (59.5%)		
Wound dehiscence	No	92 (82.9%)		72 (80.9%)		164 (82%)		0.72
	Yes	19 (17.1%)		17 (19.1%)		36 (18%)		
Intrabdominal abscess	No	99 (89.2%)		77 (86.5%)		176 (88%)		0.56
	Yes	12 (10.8%)		12 (13.5%)		24 (12%)		
Anastomotic/stump leak	No	64 (85.9%)		61 (100%)		125 (95.4%)		**0.006**
	Yes	9 (14.1%)		0 (0%)		9 (4.6%)		
Re-operation	No	109 (98.2%)		83 (93.3%)		192 (96%)		0.08
	Yes	2 (1.8%)		6 (6.7%)		8 (4%)		
		**Mean**	**SD**	**Mean**	**SD**	**Mean**	**SD**	
Age (years)		63.9	15	66.6	14.6	65.1	14.8	0.21
BMI		27	3.8	27.5	3.6	27.2	3.7	0.4
Length of stay (Days)		8.9	4.7	13.9	8.0	11.1	6.8	**<0.001**
Charlson comorbidity index (CCI)		3.4	1.2	2.9	1.3	3.1	1.2	0.23

**Table 2:** Repeated measures ANOVA of hematological laboratory tests. Comparison of test results in total and in groups of infectious (Inf) and no-infections (N/I) cases. *p* values below 0.05 are highlighted in bold format.

Laboratory Test		Day 1		Day 2		Day 3		df1. df2	F	*p*
		Mean	SD	Mean	SD	Mean	SD			
WBCs (10^3^)	Total	11.8	4.5	10.9	3.9	9.7	3.3	2. 588	24.28	**<0.001**
	N/I	11.9	4.4	10.7	3.9	9.5	3.	3. 585	0.86	0.46
	Inf	11.7	4.5	11	4	9.9	3.4			
CRP (mg/dL)	Total	11.55	8.33	17.53	8.92	16.47	8.42	2. 398	66.73	**<0.001**
	N/I	7.99	6.06	11.76	7.24	10.76	6.01	2. 396	7.24	**0.001**
	Inf	13.98	8.79	21.46	7.75	20.35	7.59			
ALB (g/dL)	Total	3.3	0.5	3.2	0.4	3.1	0.4	2. 398	99.66	**<0.001**
	N/I	3.5	0.4	3.4	0.4	3.3	0.3	2. 396	3.13	0.052
	Inf	3.2	0.5	3.0	0.4	2.9	0.4			
Transferrin (mg/dL)	Total	171.5	44.8	158.6	41.2	150.9	37.7	2. 220	76.11	**<0.001**
	N/I	182.8	43.7	172.7	41.9	163.0	37.2	2. 218	76.05	0.27
	Inf	160.7	43.5	145.3	36.2	139.4	34.7			
CRP/ALB	Total	3.7	3.0	5.8	3.2	5.6	3.1	2. 398	72.19	**<0.001**
	N/I	2.4	2.0	3.6	2.3	3.3	1.9	2. 396	10.97	**<0.001**
	Inf	4.6	3.2	7.3	2.9	7.1	2.9			
CRP/Trans	Total	0.07	0.06	0.11	0.07	0.11	0.07	2. 220	39.36	**<0.001**
	N/I	0.05	0.07	0.08	0.06	0.07	0.04	2. 218	15.12	**<0.001**
	Inf	0.08	0.06	0.15	0.07	0.15	0.07			

**Table 3:** Comparison of binary and continues variables (COPD chronic obstructive pulmonary disease, BMI body mass index, CAR C-reactive protein to albumin ratio, and CTR C-reactive protein to transferrin ratio) between patients with or without postoperative complications. *p* * Pearson’s χ^2^, *p* **: Mann–Whitney *p*. *p* values below 0.05 are highlighted in bold format.

Postoperative Complications				
Parameter		No	Yes	*p* *
		N (%)	N (%)	
Sex	Female	44 (54.3%)	42 (35.3%)	**0.008**
	Male	37 (45.7%)	77 (64.7%)	
Type of study	Prospective	54 (66.7%)	57 (47.9%)	**0.009**
	Retrospective	27 (33.3%)	62 (52.1%)	
Elective operation	No	52 (64.2%)	46 (38.7%)	**<0.001**
	Yes	29 (35.8%)	73 (61.3%)	
Age (years)	≤40	5 (6.2%)	7 (5.9%)	0.34
	41–50	3 (3.7%)	12 (10.1%)	
	51–60	10 (12.3%)	19 (16.0%)	
	61–70	25 (30.9%)	37 (31.1%)	
	71–80	23 (28.4%)	32 (26.9%)	
	81+	15 (18.5%)	12 (10.1%)	
Hypertension	No	35 (43.2%)	50 (42.0%)	0.87
	Yes	46 (56.8%)	69 (58.0%)	
Diabetes type II	No	61 (75.3%)	86 (72.3%)	0.63
	Yes	20 (24.7%)	33 (27.7%)	
COPD	No	67 (82.7%)	97 (81.5%)	0.83
	Yes	14 (17.3%)	22 (18.5%)	
Chronic renal disease	No	75 (92.6%)	106 (89.1%)	0.41
	Yes	6 (7.4%)	13 (10.9%)	
Cancer	No	40 (49.4%)	58 (48.7%)	0.93
	Yes	41 (50.6%)	61 (51.3%)	
Metastatic cancer	No	75 (92.6%)	111 (93.3%)	0.95
	Yes	6 (7.4%)	8 (6.7%)	
		**Mean (SD)**	**Mean (SD)**	*p* **
Age (years)		67.16 (14.61)	63.76 (14.86)	0.09
BMI (Kg/m^2^)		26.98 (3.4)	27.37 (3.89)	**<0.001**
Post Op CRP DAY2		11.76 (7.24)	21.46 (7.75)	**<0.001**
Post Op ALB DAY2		3.37 (0.36)	3 (0.41)	**<0.001**
Post Op WBCs DAY2		10.54 (3.86)	10.99 (3.97)	0.35
CAR DAY 2		3.59 (2.32)	7.31 (2.89)	**<0.001**
CTR DAY 2		0.08 (0.06)	0.15 (0.07)	**<0.001**
CTR DAY2 × 100		7.56 (6.01)	14.92 (6.96)	**<0.001**

**Table 4:** Results of multiple logistic regression analysis (ORs with 95% confidence intervals) using presence of postoperative septic complications) and demographic, laboratory, and chronic disease variables (OR odds ratio, LL lower limit, UL upper limit, COPD chronic obstructive pulmonary disease, BMI body mass index, CAR C-reactive protein to albumin ratio, and CTR C-reactive protein to transferrin ratio). 95%LL: lower limit of OR, 95%UL: upper limit of OR. *p* values below 0.05 are highlighted in bold format.

**Postoperative Septic Complications = Model Enter**				
**Parameter**	**OR**	**95%LL**	**95%UL**	***p***
Age (years)	0.98	0.94	1.01	0.17
BMI (Kg/m^2^)	0.90	0.79	1.02	0.11
Female sex	0.23	0.08	0.68	**0.008**
Elective operation	0.86	0.29	2.54	0.78
Hypertension	1.07	0.45	2.54	0.87
Diabetes type II	1.52	0.61	3.78	0.37
COPD	1.20	0.37	3.91	0.76
Chronic renal disease	0.99	0.19	5.25	0.99
Presence of cancer	1.70	0.69	4.21	0.25
Post-op TRF DAY2	1.01	1.00	1.03	0.14
Post-op ALB DAY2	0.05	0.01	0.33	**0.002**
CTR DAY 2 × 100	1.23	1.10	1.39	**0.000**
CTR DAY 3 × 100	1.40	1.21	1.61	**<0.001**
CAR DAY 2	1.73	1.32	2.29	**<0.001**
CAR DAY 3	2.02	1.63	2.51	**0.000**
**Postoperative septic complications = Model Forward**				
**Forward**	**OR**	**95%LL**	**95%UL**	***p***
Female sex	0.26	0.09	0.71	**0.009**
Post-op ALB DAY2	0.12	0.03	0.52	**0.005**
CTR DAY 2 × 100	1.18	1.07	1.29	**<0.001**
CTR DAY 3 × 100	1.42	1.24	1.63	**<0.001**
CAR DAY 2	1.68	1.31	2.17	**<0.001**
CAR DAY 3	2.02	1.65	2.48	**<0.001**

## Data Availability

The raw data supporting the conclusions of this article will be made available from the corresponding author without undue reservation.

## Supplementary Materials

The following supporting information can be downloaded at: https://www.mdpi.com/article/10.3390/jcm14124341/s1. Table S1. Type of operation (LAR Low anterior resection, APE abdomino-perineal excision), Table S2. Patients’ chronic diseases and cancer rates (COPD chronic obstructive pulmonary disease, N/A not applicable, n number), Table S3. Incidence of septic events per type of surgery (LAR Low anterior resection, APE abdomino-perineal excision), Table S4. Correlation of pre-operative ALB levels with SSI incidence (ALB albumin, SSI surgical site infections, OR odds ratio, CI confidence intervals).
