# Supplementary material for: Evaluation of the Diagnostic and Predictive Significance of Postoperative C-Reactive Protein to Transferrin or Albumin Ratio in Identifying Septic Events Following Major Abdominal Surgery
Source: J Clin Med. 2025 Jun 18;14(12):4341. doi: 10.3390/jcm14124341 (PMC12194131; doi:10.3390/jcm14124341)
Supplement: Supplementary file 1 [file jcm-14-04341-s001.zip › jcm-3677648-supplementary.pdf]

Journal of Clinical Medicine

MDPI

Supplementary Table S1. Type of operation (LAR Low anterior resection, APE abdomino-perineal excision)

Type of Study				
	**Prospective**	**Retrospective**	**Total**	
	N(%)	N (%)	N (%)	**p**
Hartmann's Sigmoidectomy	10 (9%)	6 (6.7%)	16 (8%)	0.65
Sigmoidectomy (with anasotmosis)	7 (6.3%)	4 (4.5%)	11 (5.5%)	
LAR	5 (4.5%)	5 (5.6%)	10 (5%)	
APE	4 (3.6%)	4 (4.5%)	8 (4%)	
Left Hemicolectomy	3 (2.7%)	6 (6.7%)	9 (4.5%)	
Right Hemicolectomy	17 (15.3%)	19 (21.3%)	36 (18%)	
Open Cholecystectomy	11 (9.9%)	4 (4.5%)	15 (7.5%)	
Abdominal Wall Hernia Repair	10 (9%)	7 (7.9%)	17 (8.5%)	
Perforated Peptic Ulcer Repair	10 (9%)	6 (6.7%)	16 (8%)	
Enterectomy	12 (10.8%)	15 (16.9%)	27 (13.5%)	
Laparotomy	16 (14.4%)	11 (12.4%)	27 (13.5%)	
Appendectomy	6 (5.4%)	2 (2.2%)	8 (4%)	

Supplementary Table S2. Patients' chronic diseases and cancer rates (COPD chronic obstructive pulmonary disease, N/A not applicable, n number)

Type of Study					
		**Prospective**	**Retrospective**	**Total**	
		N (%)	N (%)	N (%)	**p**
Hypertension	No	30 (35.3%)	55 (64.7%)	85 (42.5%)	**<0.001**
	Yes	81 (70.4%)	34 (29.6%)	115 (57.5%)	
Diabetes Type II	No	78 (53.1%)	69 (46.9%)	147 (73.5%)	0.25
	Yes	33 (62.3%)	20 (37.7%)	53 (26.5%)	
COPD	No	89 (54.3%)	75 (45.7%)	164 (82%)	0.45
	Yes	22 (61.1%)	14 (38.9%)	36 (18%)	
Chronic Renal Disease	No	101 (55.8%)	80 (44.2%)	181 (90.5%)	0.79
	Yes	10 (52.6%)	9 (47.4%)	19 (9.5%)	
Cancer	No	61 (62.2%)	37 (37.8%)	98 (49%)	0.06
	Yes	50 (49%)	52 (51%)	102 (51%)	
Metastatic Cancer	No	103 (55.4%)	83 (44.6%)	186 (93%)	0.9
	Yes	8 (57.1%)	6 (42.9%)	14 (7%)	

Inflammatory Bowel Disease	No	111 (55.5%)	89 (4.5%)	200 (100%)	N/A
	Yes	0 (0.0%)	0 (0.0%)	0 (0.0%)	

Supplementary Table S3. Incidence of septic events per type of surgery (LAR Low anterior resection, APE abdomino-perineal excision)

		Wound Infection	Wound dehiscence	Intrabdominal Abscess	Anastomotic Leak	Re-operation
Hartmann's Sigmoidectomy	Prospective	7/10	5/10	4/10	4/10	0/10
	Retrospective	5/6	2/6	1/6	0/6	1/6
Sigmoidectomy (with anasotmosis)	Prospective	3/7	1/7	1/7	1/7	0/7
	Retrospective	2/4	0/4	1/4	0/4	0/4
LAR	Prospective	1/5	0/5	0/5	0/5	0/5
	Retrospective	2/5	1/5	0/5	0/5	0/5
APE	Prospective	4/4	0/4	0/4	0/4	0/4
	Retrospective	3/4	1/4	1/4	0/4	0/4
Left Hemicolectomy	Prospective	0/3	0/3	0/3	0/3	0/3
	Retrospective	2/6	0/6	0/6	0/6	1/6
Right Hemicolectomy	Prospective	9/17	3/17	2/17	1/17	1/17
	Retrospective	16/19	5/19	2/19	0/19	1/19
Open Cholecystectomy	Prospective	5/11	3/11	2/11	0/11	0/11
	Retrospective	4/4	1/4	1/4	0/4	1/4
Abdominal Wall Hernia Repair	Prospective	2/10	0/10	0/10	0/10	0/10
	Retrospective	4/7	1/7	1/7	0/7	1/7
Perforated Peptic Ulcer Repair	Prospective	6/10	2/10	1/10	2/10	1/10
	Retrospective	3/6	1/6	1/6	0/6	0/6
Enterectomy	Prospective	9/12	2/12	2/12	1/12	0/12
	Retrospective	12/15	3/15	2/15	0/15	1/15
Laparotomy	Prospective	7/16	2/16	0/16	0/16	0/16
	Retrospective	7/11	2/11	1/11	0/11	0/11
Appendectomy	Prospective	4/6	1/6	0/6	0/6	0/6
	Retrospective	2/2	0/2	1/2	0/2	0/2

Supplementary Table S4. Correlation of pre-operative ALB levels with SSI incidence (ALB albumin, SSI surgical site infections, OR odds ratio, CI confidence intervals)

Preoperative ALB Range (g/dL)	Patients (n)	SSI Incidence n (%)	OR(95% CI)	p
ALB ≤2.5	2	1(50%)	1.22 (0.07, 20.55)	0,054
2.5 <ALB≤3	9	8(88,9%)	9.74 (1.13, 83.68)	
3< ALB ≤3.5	16	12(75%)	3.65 (1.04, 12.86)	
3.5<ALB ≤4	33	16(48,5%)	(0.48, 2.76)	
ALB >4	51	23(45,1%)		
Total	111	60 (54.1%)		
